# Proteins involved in the biosynthesis of lipophosphoglycan in *Leishmania*: a comparative genomic and evolutionary analysis

**DOI:** 10.1186/s13071-020-3914-9

**Published:** 2020-01-30

**Authors:** Lucas Gentil Azevedo, Artur Trancoso Lopo de Queiroz, Aldina Barral, Luciane Amorim Santos, Pablo Ivan Pereira Ramos

**Affiliations:** 1 Instituto Gonçalo Moniz, Fundação Oswaldo Cruz (FIOCRUZ), Salvador, Bahia, Brazil; 2 Escola Bahiana de Medicina e Saúde Pública, Salvador, Bahia Brazil; 3 Post-graduate Program in Biotechnology and Investigative Medicine, Instituto Gonçalo Moniz, Salvador, Bahia Brazil; 4 Universidade Federal da Bahia, Salvador, Bahia Brazil; 5 Instituto de Investigação em Imunologia (iii-INCT), São Paulo, São Paulo Brazil

**Keywords:** Genome mining, *Leishmania*, Lipophosphoglycan, Phylogenomics, Trypanosomatids

## Abstract

**Background:**

*Leishmania* spp. are digenetic parasites capable of infecting humans and causing a range of diseases collectively known as leishmaniasis. The main mechanisms involved in the development and permanence of this pathology are linked to evasion of the immune response. Crosstalk between the immune system and particularities of each pathogenic species is associated with diverse disease manifestations. Lipophosphoglycan (LPG), one of the most important molecules present on the surface of *Leishmania* parasites, is divided into four regions with high molecular variability. Although LPG plays an important role in host-pathogen and vector-parasite interactions, the distribution and phylogenetic relatedness of the genes responsible for its synthesis remain poorly explored. The recent availability of full genomes and transcriptomes of *Leishmania* parasites offers an opportunity to leverage insight on how LPG-related genes are distributed and expressed by these pathogens.

**Results:**

Using a phylogenomics-based framework, we identified a catalog of genes involved in LPG biosynthesis across 22 species of *Leishmania* from the subgenera *Viannia* and *Leishmania*, as well as 5 non-*Leishmania* trypanosomatids. The evolutionary relationships of these genes across species were also evaluated. Nine genes related to the production of the glycosylphosphatidylinositol (GPI)-anchor were highly conserved among compared species, whereas 22 genes related to the synthesis of the repeat unit presented variable conservation. Extensive gain/loss events were verified, particularly in genes *SCG1-4* and *SCA1-2.* These genes act, respectively, on the synthesis of the side chain attached to phosphoglycans and in the transfer of arabinose residues. Phylogenetic analyses disclosed evolutionary patterns reflective of differences in host specialization, geographic origin and disease manifestation.

**Conclusions:**

The multiple gene gain/loss events identified by genomic data mining help to explain some of the observed intra- and interspecies variation in LPG structure. Collectively, our results provide a comprehensive catalog that details how LPG-related genes evolved in the *Leishmania* parasite specialization process.
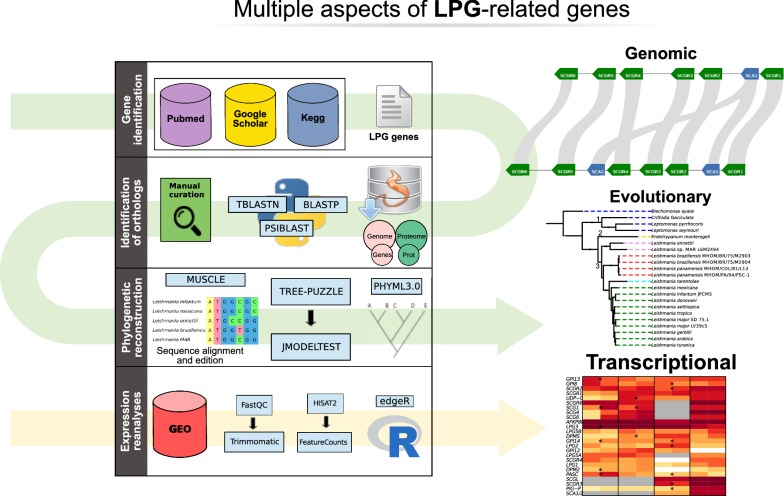

## Background

The *Leishmania* spp. are protozoan parasites belonging to the family Trypanosomatidae. These microorganisms are digenetic, spending a portion of their life-cycle in an invertebrate host and another part in a vertebrate host. During these stages, the morphology and metabolism of the parasite changes drastically. Mammalians are one of the vertebrate hosts inhabited by the parasite, and, when bitten by an infected sand fly vector (Diptera: Phlebotominae), *Leishmania* promastigotes are inoculated into the blood pool in the mammalian hostʼs dermis along with sand fly saliva once the mosquito takes a blood meal [1, 2]. Once inside the host, parasites, which are phagocytosed by immune cells, such as macrophages and neutrophils, assume a rounded shape and transit into amastigote forms, which are highly adapted to the phagolysosomal milieu [3, 4]. With regard to the sand fly vector (e.g. *Lutzomyia* spp. and *Phlebotomus* spp.), *Leishmania* parasites inhabit the midgut and differentiate into promastigote. Thereafter, the parasites migrate to the pharyngeal valve. The specific binding site and migration course in the phlebotomine gut varies in accordance with parasite species. For instance, species of the subgenus *Viannia*, e.g. *L.* (*Viannia*) *braziliensis*, remain in the hindgut and then migrate to the midgut, while species of the subgenus *Leishmania*, e.g. *L.* (*Leishmania*) *infantum,* develop in the posterior midgut and subsequently reach the anterior midgut [5].

The manifestations caused by *Leishmania* infection are collectively termed leishmaniasis and are responsible for serious public health problems in many poor-income countries [6]. The resulting clinical presentation is dependent on *Leishmania* species, as well as parasite interplay with the host immune system. In humans, leishmaniasis manifestations range from cutaneous, mucocutaneous, or visceral. Localized cutaneous leishmaniasis (LCL) is characterized by ulcers of different sizes at the site of inoculation, while mucocutaneous leishmaniasis (ML) affects the skin and mucosae. Visceral leishmaniasis (VL) is the most severe and life-threatening form, where the parasite diffuses through the bloodstream, reaching essential organs, such as the liver and spleen, and eventually bone marrow [7–11].

Lipophosphoglycans (LPGs), the main glycoconjugate found on the surface membranes of *Leishmania* parasites, are constitutively expressed in promastigote forms, while low expression levels are seen in amastigotes [12]. LPG is also a virulence factor that participates directly in parasite attachment and establishment within the sand fly midgut, where vector lectins interact with the lateral chains of LPG repetition units [13]. This lipophosphoglycan can also delay lysosome fusion with the phagosome and disrupt the assembly of NADPH oxidase on the phagosomal membrane [14].

The LPGs are composed of four elements: (i) a phosphatidylinositol anchor; (ii) a conserved (core) glycan group consisting of β-linkages between galactose, mannose, and glucosamine; (iii) phosphoglycan repeating (PG) units (Gal β1,4 Man α1-PO_4_), which are, in some cases, associated with side chains; and (iv) a neutral oligosaccharide called cap [15, 16]. Variations in the composition and length of these structures, particularly the side chain and cap regions, are evidenced among parasite species and morphological forms (amastigotes or promastigotes) [17]. Although previous reports have described several genes related to LPG biosynthesis, the pathway as a whole has not been entirely elucidated and is known to involve multiple metabolic sub-routes [18–21].

The present study endeavoured to identify the repertoire of genes involved in LPG production by *Leishmania* using a comprehensive data mining approach combining genomic, transcriptomic and high-quality primary literature searches. All findings were supported by extensive manual curation and annotation of genomic search results. Moreover, the evolutionary relationships between the identified genes were evaluated. Our results allow for an improved understanding regarding how LPG-related genes were shaped during the evolutionary diversification of *Leishmania* parasites to different vectors, disease manifestations and geographical ranges.

## Methods

**Fig. 1:**
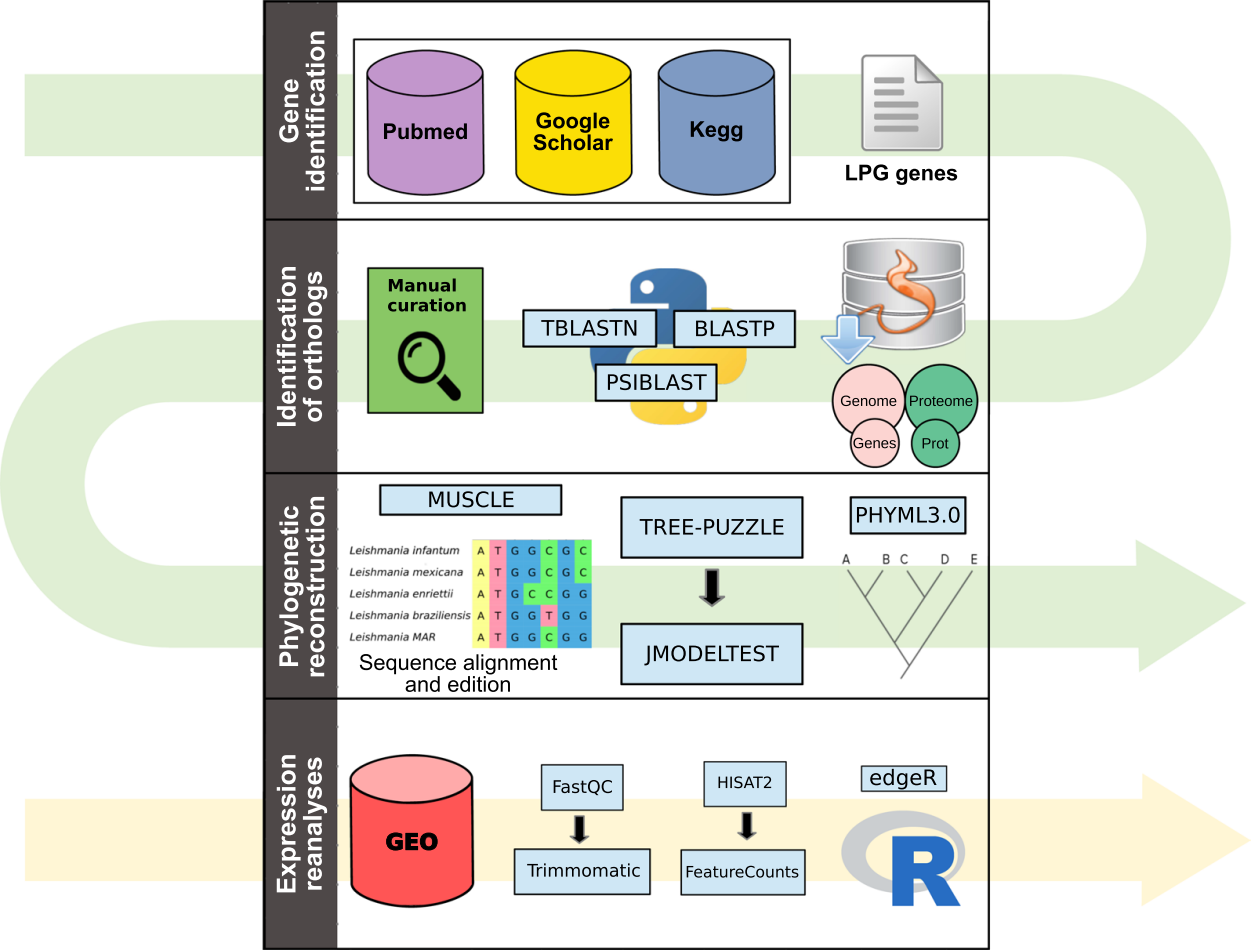
Scheme summarizing the steps of the methodology. In order to determine the catalog of genes related to LPG synthesis, searches were performed in three databases (primary literature (NCBI/PubMed; Google Scholar) and metabolic information (KEGG)). To establish orthology relationships, the nucleotide and amino acid sequences of these genes were obtained from TriTrypDB along with 22 genomes and proteomes of trypanosomatid species. To evaluate the similarity between these sequences, three variants of the BLAST algorithm were used, and the execution of these sequences was performed using a custom-tailored Python script and subsequently curated, yielding groups of ortholog genes across species. The phylogenetic reconstruction was performed from the alignment of the groups using Muscle, and the evaluation of phylogenetic signal in the groups was performed with Tree-Puzzle. The most informative evolutionary model was estimated with jModelTest2 and a maximum likelihood phylogenetic tree was constructed using PHYML

The current study employed multiple computational techniques in order to derive a set of LPG-related genes across various trypanosomatids. Particularly, we used genomic data mining, phylogenetic analyses and re-evaluation of selected RNA-seq datasets, which are summarized in Figure 1, and further described in the following sections.

### Identification of the catalog of genes related to LPG biosynthesis from the primary literature

To determine the set of genes related to LPG synthesis, we used the primary literature (articles indexed in PubMed and Google Scholar) in which experimentally characterized protein sequences were reported. Textual searches were performed using Boolean operators and the keywords: “LPG biosynthesis” AND “*Leishmania*”. In addition, searches using the Kyoto Encyclopedia of Genes and Genomes (KEGG; http://www.genome.jp/kegg/) database [22]⁠ were performed, particularly using the pathway maps related to the GPI anchor synthesis (KEGG accession map00563). TriTrypDB (http://tritrypdb.org) [23] was also used as an information source, facilitating the retrieval of genes, genomes and proteomes from the organisms used in this study. This allowed the identification of gene names, gene families, and molecular functions that relate to the synthesis of LPG in these organisms. Once identified, the genes were downloaded from TriTrypDB using *Leishmania major* Friedlin as a reference organism [23] along with the complete genomes and proteomes (when available) of the species included in the study (Additional file 1: Table S1). A total of 27 organisms were included, most of them (*n* = 22) from the genus *Leishmania* (excluding *L. amazonensis* due to the absence of a chromosome-level genome assembly). Also studied were the genomes of other non-*Leishmania* trypanosomatids, including *Blechomonas ayalai* (*n* = 1), *Crithidia fasciculata* (*n* = 1), *Leptomonas* spp. (*n* = 2), and *Endotrypanum monterogeli* (*n* = 1). Their inclusion allowed to study LPG-related genes from an evolutionary standpoint given the more basal position of these organisms. Genomes of *Trypanosoma* spp. were not included in the study due to the low similarity of the majority of studied LPG-related proteins in these organisms, which is in agreement with the reported lack of lipophosphoglycan in their surface, despite the presence of other surface molecules such as variant surface glycoproteins (VSGs) [24].

### Inference of LPG orthologs across multiple trypanosomatids

From the high-quality gene set determined in the previous step, sequence similarity searches were performed in locally created BLAST databases [25] comprising proteome or genome sequences from the studied organisms, using *L. major* Friedlin genes as queries from the previous step. For this purpose, the BLASTP (protein search against proteome), TBLASTN (protein search against genome), and PSI-BLAST (search of distant proteins against proteome iteratively) suite of algorithms included in the BLAST+ 2.7.1 package [26] were used to conduct similarity searches. Particularly, phylogenetically more distant orthologs (which have accumulated more changes, for example, or are present in organisms with more divergent evolutionary history) were retrieved using PSI-BLAST with up to five iterations. Orthology was defined by visually inspecting the alignments and considering the following properties: sequence identity, coverage (*minLrap* and *maxLrap*), and genomic context conservation (synteny). Coverage and identity criteria of 60% and 80%, respectively, were used as guides during the manual curation, albeit these cut-offs were relaxed for some proteins when comparing more distant organisms with evidence such as strong synteny support and domain conservation. Custom Python scripts (http://www.python.org) were written to automatically perform large-scale BLAST analyses, calculate coverage statistics from the text outputs, perform initial filtering steps that classified each queried protein as present or absent in an organism, and prepare tabular outputs. These results were then manually curated. For *SCG* family genes, which present extensive sequence conservation and locate in telomeric regions [27], we additionally employed the Mauve algorithm v2.4.0 [28] to resolve their position and identity, which helped to assign a correct gene annotation. Coverage statistics were calculated as follows: $minLrap = \frac{length\, of\, the\, match}{{min\left( {L_{1} ,L_{2} } \right)}},maxLrap = \frac{length\, of\, the\, match}{{max\left( {L_{1} ,L_{2} } \right)}}$, where *L*_*1*_ and *L*_*2*_ are the lengths of two compared proteins.

### Reconstruction of maximum likelihood phylogenetic trees

The phylogenetic reconstruction of genes identified in the previous step was performed to infer the evolutionary history and within-family relationships between the different species. For this analysis we concatenated genes according to each LPG structural region. Genes that had a high level of conservation (having a single ortholog in each species) were divided into groups considering the structure of the LPG in which it exerted its metabolic function (anchor genes or repeat unit genes) and later concatenated into single sequences corresponding to each species. Gene multiple alignment was carried out using Muscle version 3.8.31 [29] followed by manual curation of the alignments [30]. Phylogenetic signal was determined by Tree-Puzzle 5.2 [31], with the likelihood mapping approach. For the maximum likelihood tree identification, the PhyML [32] version 3.3.20180621 was used with the model GTR+I+G, selected by employing the Akaikeʼs information criterion within jModelTest 2.1.10 program [33]. The tree support indexes were determined by bootstrap with 1000 replicates. The trees were edited using FigTree v1.4.3 (http://tree.bio.ed.ac.uk/software/figtree/) and iTol [34].

### Evaluation of the expression of LPG-related genes

In order to find transcriptional evidence for the identified genes, a reanalysis of previously published transcriptomic datasets deposited in the Sequence Read Archive (SRA/NCBI; http://www.ncbi.nlm.nih.gov/sra) was performed. Datasets were selected based on the comparison of different *Leishmania* life stages, and for which transcriptome profiling using RNA-seq methodology was available [35–37]. These included promastigote and amastigote samples for *L. mexicana* [36] and *L. donovani* [35] in axenic cultures; and *L. major* and *L. amazonensis* during metacyclic promastigote stage as well as intracellularly 4 h and 48 h post-macrophage infection [37]. The sequence accessions of these samples are listed in Additional file 2: Table S2. To avoid methodology bias, all sequences were reanalyzed using the same integrated pipeline, as follows: first, FastQC was used to perform read quality-control (QC) [38]. Then, filtering of low-quality sequences and adapter trimming were performed using Trimmomatic v. 0.38 (parameters: LEADING:3; TRAILING:3; SLIDINGWINDOW:4:20; and MINLEN:70) [39]. HISAT2 v. 2.1.0 [40] was used to map the reads that passed QC against the genomes of *L. donovani* BPK282A1, *L. major* Friedlin and *L. mexicana* MHOM/GT/2001/U1103, which were obtained from TriTrypDB version 42. Due to the low quality of the *L. amazonensis* MHOM/BR/71973/M2269 genome, reads from this species were mapped against the genome of *L. mexicana* MHOM/GT/2001/U1103. FeatureCounts v. 1.6.3 was used to obtain a gene count table [41], and edgeR was employed to perform normalization of the expression data using the trimmed mean of M-values method (TMM) [42]. TMM-normalized counts per million (cpm) values were obtained for each gene, and to facilitate comparison across different experiments and conditions, the expressional level of each LPG-related gene was discretized into deciles. Non-parametric tests were used to evaluate differences across life forms and parasite species using as input the normalized cpm values. For two-sample comparisons, the Mann–Whitney test was used, while for three-sample comparisons the Kruskal-Wallis test was employed followed by Dunnʼs *post-hoc* test, and significance was considered when *P*-values were less than 0.05.

## Results and discussion

### Building a catalog of genes related to lipophosphoglycan biosynthesis in trypanosomatids

**Fig. 2:**
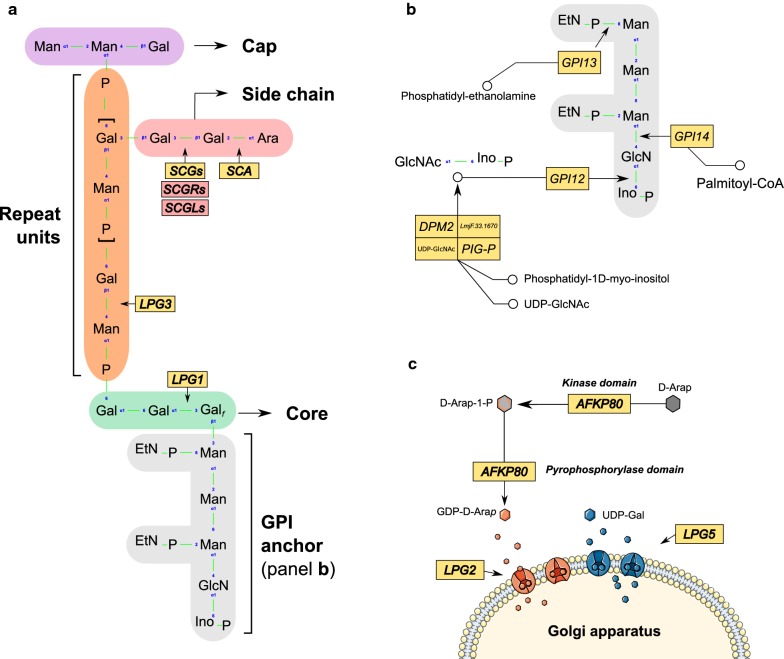
A metabolic map related to LPG synthesis in *Leishmania*. **a** The LPG molecule, composed of four main parts (GPI anchor, core, repeat unit and cap). The branched polysaccharide residues that usually compose this molecule are shown, and the sites where LPG-related gene products act for the synthesis of this molecule are displayed together with the gene name in yellow boxes. *SCGR/L* family genes proposed to participate in side chain formation are highlighted in salmon boxes. Genes that are involved in the synthesis of the repeat unit and core regions are shown, while those that form the GPI anchor appear in **b**. **c** The conversion of d-arap into GDP-d-Arap by the dual kinase/pyrophosphorylase activity of *AFKP80*, and the transport of GDP-d-Arap and UDP-Gal into the Golgi apparatus. *Abbreviations*: Ara, arabinose; d-Arap, d-arabinopyranose; EtN, ethanolamine; Gal, galactose; Gal_f_, galactosylfuranose; GlcN, glucosamine; GlcNAc, N-acetyl glucosamine; Ino, inositol; Man, mannose

The first step in our study was to perform searches in primary literature databases for genes associated with LPG synthesis. As a result, we identified 9 genes associated with glycosylphosphatidylinositol (GPI)-anchor biosynthesis (belonging to the anchor and core regions) and 22 genes related to the repeat unit assembly, totaling 31 genes. The selection of genes that were studied in the present study participate in diverse biochemical reactions that lead to the synthesis of LPG. These include the addition of molecules into the core and repeat unit (Fig. 2a), GPI anchor (Fig. 2b), and transport of these molecules (Fig. 2c). Events of gain/loss of LPG genes observed across the compared trypanosomatids could lead to diversity in the structure of LPG in these organisms.

The search for these genes used as a query the *Leishmania major* Friedlin genome, as this strain offers superior quality of sequencing and annotation data within the reported trypanosomatids. The accession numbers, genomic location, and protein products for the 31 LPG-related genes are shown in Additional file 3: Table S3. These genes were queried using multiple variations of the BLAST algorithm within the genomes/proteomes of the studied trypanosomatids. The TriTrypDB database was the primary information source, and related metadata (including stable accessions, genome size, G + C%, and gene count) are available in Additional file 1: Table S1. Sequence alignments were thoroughly manually curated in order to evaluate duplications, frameshifts and any other possible rearrangements. Figure 1 summarizes the data processing workflow and methodology.

**Fig. 3:**
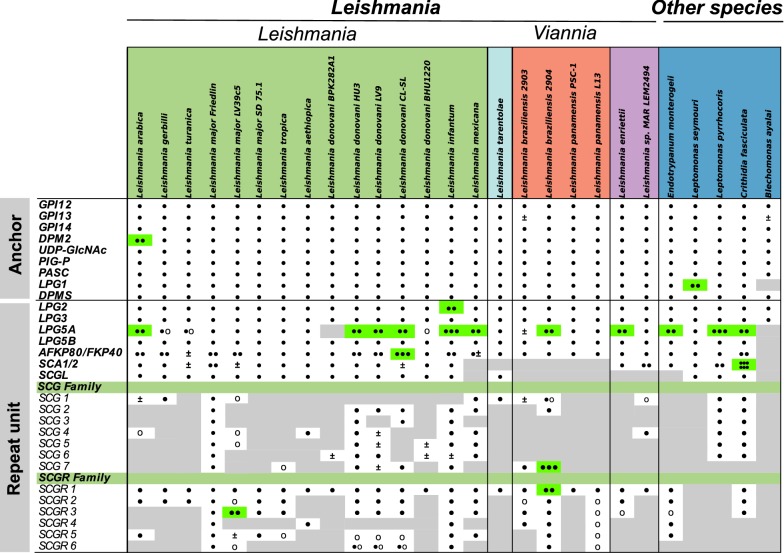
A catalog of trypanosomatid genes involved in the synthesis of LPG. The genes in each row are grouped by their involvement in the synthesis of molecules in either the anchor or repetition regions of LPG. *FKP40* and *AFKP80*, as well as *SCA1* and *SCA2* genes were jointly considered because of their high degree of sequence conservation that hampers their differentiation by sequence analysis alone. Species belonging to the *L. enrietti* complex are marked in purple in the top panel, and those belonging to *Sauroleishmania* in light blue. The subgenera *Leishmania* and *Viannia* are shown in green and red, respectively. For retrieved sequences, the following symbols were used to indicate the presence of an ortholog for the gene (●), frameshift within the gene (±), and sequences with a partial alignment (o). Events of gene gain and loss are represented in green and gray, respectively. The sequences used as query for the BLAST searches were from *L. major* Friedlin. Presentation of the data was inspired by the previous genome-based work of Arcá et al. [73]

Once a curated dataset was obtained, we summarized the repertoire of LPG-related orthologs identified by our whole-genome mining strategy in the 26 queried organisms compared to *L. major* Friedlin (Fig. 3). A total of 549 proteins were recovered, 16 sequences were re-annotated (from being previously annotated as hypothetical proteins; listed in Additional file 4: Table S4), and 22 open reading frames (ORFs) were newly predicted as a result of our analyses (Additional file 4: Table S4). Information on these sequences, including protein products and corresponding ORF coordinates, are available in Additional file 5: Table S5. The 31 LPG-related genes were then grouped based on the metabolic processes in which they participate, i.e. either the synthesis of the GPI anchor region or the repeat unit. The following sections provide further details regarding the results related to each synthesis region.

### Genes involved in GPI molecule biosynthesis are mostly conserved and well-distributed among trypanosomatids

The repertoire of LPG-related genes in Figure 3 revealed a high degree of conservation in genes related to GPI-anchor biosynthesis among the compared parasites. This stability in gene content was not unexpected, given that the GPI-anchor structure is well-conserved across species and represents the building block upon which a series of sugar molecules are anchored, such as glycosylinositolphospholipids (GIPLs) and GP63 [43–45]. Despite this sequence conservation, some exceptions were observed. For instance, in the genome of *L. braziliensis* MHOM/BR/75/M2903, the *GPI13* gene ortholog (coding for ethanolamine phosphate transferase 3) presented a frameshift generated by a deletion at position 102,951 of chromosome 24 that gave rise to two ORFs: LBRM2903_240008400, with a length of 1365 bp, and LBRM2903_240008500, located 63 bp downstream with a size of 1497 bp that starts at position 103,018. Both had nucleotide identities of approximately 78% to the *L. major* query. Considering this similarity, the degree of conservation and the importance of this gene, as well as the presence of the complete ortholog in the related strain MHOM/BR/75/M2904 (Fig. 3), it is plausible that this frameshift likely stemmed from a sequencing error. The sequencer used to obtain this genome, Roche 454 FLX, supports this hypothesis, given its proneness to insertion/deletion (InDel)-type errors. Another exception is our result for the *LPG1* gene, which lacked a corresponding ortholog in the genome of *Blechomonas ayalai* (a monoxenous species of flea parasites), which also appeared duplicated in *Leptomonas seymouri*, a monoxenous parasite capable of infecting mammals under specific circumstances (e.g. an immunocompromised host) [46]. This gene is vitally important to the structure of LPG, as it encodes a galactosylfuranose transferase responsible for the Gal_f_ (β1,3) Man binding, with specific activity in the LPG core; in its absence, the remaining structure of the LPG molecule cannot be correctly assembled. A possible explanation for the lack of *LPG1* in *B. ayalai* might be its more phylogenetically distant position from all other species included in this study. The paralogs identified in *L. seymouri* may accomplish distinct activities, similarly to what was also observed for homologs of this gene found in the genome of *L. major* Friedlin, which exert functions unrelated to LPG synthesis (e.g. LPG1G family members that participate in the production of GIPLs) [18]. The differentiation of other galactosylfuranoses from *LPG1*-like sequences is more straightforward in *Leishmania*, as these organisms possess a high degree of conservation in their genomic contexts, but this becomes more complicated when more distant species, such as *Leptomonas*, are analyzed. In this context, what could be construed as a duplication event could also represent a functionally distinct coding sequence. The fact that *LPG1* was present in almost all of the studied organisms, together with its importance to the synthesis of LPG, makes it an attractive object of study across various *Leishmania* species, as has been performed in *L. major*, *L. infantum* and *L. donovani* using knock-outs of this gene [47, 48].

**Fig. 4:**
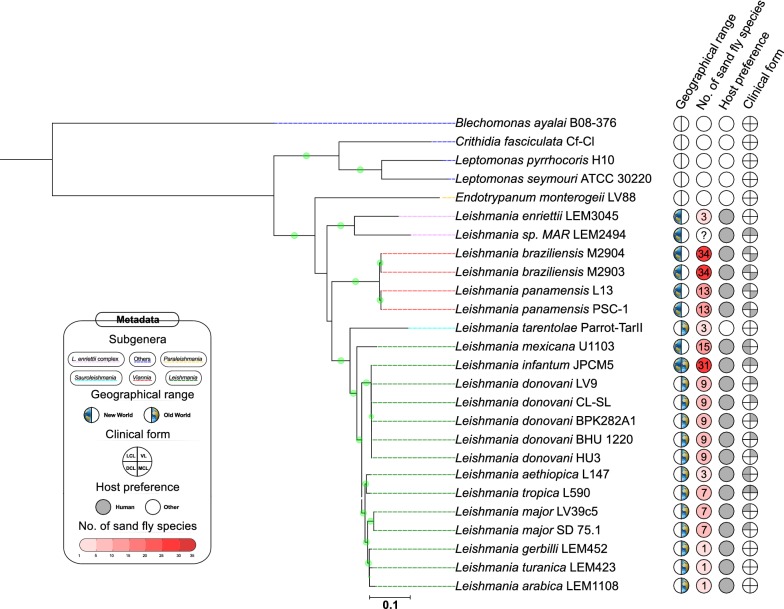
Phylogenetic maximum likelihood tree representing the evolutionary history of concatenated genes related to GPI anchor biosynthesis. Bootstrap values (1000 replications) greater than 80% are represented by a green circle in the branches of the tree. The right-hand panel classifies species according to their geographical range, number of sand fly species reported to act as vector for each parasite (at the species level), host preference and clinical form of the disease for species that are infective for humans. Colored dashes indicate the subgenus for each species, with those not classified into this taxonomic rank grouped as “Others”. The metadata were extracted from Akhoundi et al. [74]

A phylogenetic tree was constructed with all concatenated orthologs of the genes related to the synthesis of the anchor region (Fig. 4). This analysis provided an overview of the evolutionary history of GPI-anchor related genes, as this structure is required for the binding of several membrane compounds of *Leishmania*. Figure 4 also shows data on the geographical region in which each species is endemic, as well as clinical manifestations (when pathogenic) and host candidates. The clusters observed in this tree follow the subgenera classification of trypanosomatid species, while also differentiating between New and Old World *Leishmania* species. Of note, the robust bootstrap support for most of the identified clusters indicates that the choice of genes used to perform this analysis was appropriate. Within the *Euleishmania* lineage, the most basal cluster is formed by species of the *L. enriettii* complex, and this clade is further subdivided into taxa belonging to the subgenera *Viannia* and *Leishmania*. Interestingly, this phylogenetic placement is consistent with other studies that used an even higher number of genes [49], indicating that the set of GPI-anchor genes studied here recapitulate the key features of *Leishmania* evolution. It is worth noting that the three *Leishmania* species that do not cause disease (*L. arabica*, *L. gerbilli* and *L. turanica*) were grouped into a single cluster (Fig. 4). The observed evolutionary pattern was also linked to geographical range (*L. gerbilli* and *L. turanica* are found in Central Asia, South Mongolia and Iran, and *L. arabica* in Saudi Arabia) and to the number of sand fly vectors. The relatedness of this group with that of *L. major* (Fig. 4) also favors this idea, since the same vector also transmits this species. The presence of LPG-specific receptors in the sand fly midgut supports the notion that similarities among GPI-anchor genes in *Leishmania* are driven more by shared vector characteristics than by vertebrate host species preference [50].

### Genes participating in the synthesis of the repeat unit present variable conservation and extensive gain/loss events across compared species

Most of the genes related to the synthesis of the PG (Gal-Man-PO_4_) region (*LPG2*, *LPG3*, *LPG5A*, and *LPG5B*) had orthologs identified in the studied organisms, with many gene gain/loss events observed (Fig. 3). All the genes except for *LPG3* code for nucleotide-sugar transporters (NST), proteins responsible for transporting the nucleotide-sugars synthesized in the cytoplasm to the Golgi lumen or the endoplasmic reticulum, both organelles that participate in LPG assembly [21, 51]. *LPG2* encodes a GDP-mannose transporter, a key carrier for supplying the substrate necessary for PG synthesis. Knockouts of this gene in *L. donovani* and *L. major* resulted in the complete absence of PGs [19, 52]. A single copy of this gene was found in all studied organisms, exception for *L. infantum* JPCM5, for which a recently published genome re-sequencing effort involving long PacBio reads revealed the presence of two tandem gene arrays that include copies of *LPG2* [53], in contrast to the original genome sequence which harbored a single copy of this gene. This leads us to speculate that the same may be true for other *Leishmania* species, which should be considered as an important factor that may preclude attempts to develop mutants for these genes using traditional recombination techniques [54].

*LPG5A* and *LPG5B* are genes responsible, at least in *L. major*, for the transport of UDP-galactose. *LPG5A* is involved in the pathway that synthesizes the PGs present in LPG as well as its side chain, while *LPG5B* participates in the PG biosynthesis of PPG, another component of the *Leishmania* membrane. Both genes were included in our analysis because their double knockout was shown to result in a complete absence of PGs [21]. While *LPG5B* occurs as single-copy in all species in which an ortholog of this gene was identified (except for *B. ayalai*), several gain events were observed for *LPG5A*, especially in *L. infantum* (three duplications), *L. mexicana*, *L. braziliensis* MHOM/BR/75/M2904, as well as *L. donovani* genomes HU3, LV9 and CL-SL, all harboring two copies of this gene. The absence of an *LPG5A* ortholog in *L. donovani* BPK282A1 is noteworthy. Further study is warranted regarding the implications of this extensive repertoire of NST in these organisms, as *Leishmania* spp. are known for using gene dosage as a mechanism to increase transcriptional activity at both single-gene and chromosomal levels [55]. The duplication events identified in this study could be an effect of these types of mechanisms to shape variability in the membrane components of these parasites.

Another monosaccharide important to the structure of LPG is arabinose in its dexter form, with a ring containing six carbon molecules known as d-Arabinopyranose (d-Ara*p*). This molecule participates directly in parasite detachment from the sand fly midgut *via* transfer to the end of the galactose side chain [56]. When capped with d-Ara*p*, this region loses its affinity to the lectins present in the intestinal mucosa, resulting in parasite detachment and subsequent migration to the upper portion of the intestinal tract [56–58]. However, for d-Ara*p* to be inserted into this structure, it must first be associated with the GDP molecule, a process that involves piro- and phosphorylation catalyzed by the protein encoded by *AFKP80.* This complex is then transported by LPG2 to the Golgi complex, where it becomes available to arabinotransferases encoded by *SCA1* and *SCA2* [59]. A paralog of *AFKP80* (termed *FKP40*) was identified in the genome of *L. major* Friedlin with a 99.75% identity, differing by only three amino acids in the *N*-terminal located in the enzymatic domain responsible for pyrophosphorylase activity. This minor variation was sufficient to provoke the loss of its pyrophosphorylase action [59]. Due to the high degree of conservation between this gene and its paralog, our search for orthologs of *AFKP80* or *FKP40* was conservatively defined in order to identify any of their possible orthologs, which is why both genes appear collapsed in Figure 3. We observed that the majority of studied New World *Leishmania*, as well as the non-*Leishmania* trypanosomatids included, have only one of the paralogs coded in their genomes, whereas most of Old World *Leishmania* present two paralogs. Another phylogenetic tree containing the hitherto discussed genes (*LPG2*, *LPG3*, *LPG5A*, *LPG5B*, *AFKP80/FKP40*) responsible for repeat unit synthesis was constructed (data not shown), showing similar topology to that built using the GPI anchor genes (Fig. 4).

Since the arabinotransferases *SCA1* and *SCA2* are absent in several species of *Leishmania*, particularly those of the subgenus *Viannia*, e.g. *L. braziliensis* and *L. panamensis* (Fig. 3), this suggests that these species are dependent on a distinct mechanism for detachment from the gut that is not linked to arabinose. The fact that these species bind to the hindgut at the beginning of sand fly infection, and not to the midgut as seen in parasites of the subgenus *Leishmania*, may help explain the absence of these genes [1]. An exception is *L. mexicana*, since the genome of this species does not code for *SCA* representatives despite belonging to the subgenus *Leishmania* (Fig. 3). Studies to determine the existence of residues of d-Ara*p* in the glycoconjugates of the above-cited parasites, as well as their function, will help shed light on these divergent findings. In addition, *SCA/SCGL* genes could potentially function as markers of specific leishmaniasis presentations, such as VL. In Brazil, *L. infantum*, along with *L. amazonensis*, and in rare cases, *L. braziliensis*, is responsible for the visceral manifestation of leishmaniasis [60, 61], with around 3500 cases reported annually [62]. The fact that we identified *SCA* genes exclusively in *L. infantum* provides evidence that these could present a possible target for identifying the causative species in VL cases using molecular biology techniques, such as PCR. Six *SCA* orthologs were recognized in *Crithidia fasciculata* (Fig. 3), perhaps because this species bears an LPG-like membrane compound termed lipoarabinogalactan (LAG), which harbors d-galactan-bound arabinose residues in its structure [63, 64].

While gene members of the SCG/R/L family (*SCG1-7*, *SCGR1-6*, and *SCGL*), 14 in all, have the predicted function of galactosyltransferases, a study by Dobson et al. [27] found that *SCG1-4* were the only ones that exhibited β(1-3)-galactosyltransferase activity in the side chain of *L. major* Friedlin LPG. Nonetheless we included all of the genes in this family in our study, as they are likely all transcribed in stable messenger RNAs, with variable patterns of expression at different parasite life stages. Although only a few presented confirmed activity, the remaining members were also considered as having the potential to encode active glycosyltransferase enzymes [27].

**Fig. 5:**
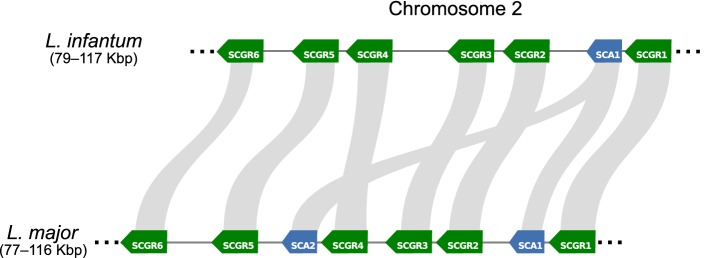
Synteny plot of *SCGR1-6* genes and *SCAs* genes between *L. major* Friedlin and *L. infantum* JPCM5 located on chromosome 2. Each gene is depicted as an arrow (where direction reflects gene orientation), and the connecting gray segments indicates sequence conservation (nucleotide identity ≥ 90%) between *L. infantum* and *L. major* genomic contexts. While *SCA1* (blue-colored) appears as a single-copy gene in *L. infantum*, located between *SCGR2* and *SCGR1*, it appears duplicated in *L. major*

In *L. major* Friedlin, the *SCG1-7* genes are distributed among multiple chromosomes (7, 21, 36, 31, 35, 25 and 2, respectively), and are always located in the telomeric region, while the genes belonging to the *SCGR* family were all found in chromosome 2 in a cluster interspersed by the *SCA1-2* genes (Fig. 5). In *L. major*, we identified the location of *SCGL* in an internal region of chromosome 14, 38 kbp distant from the right telomere; in the other *Leishmania* investigated herein, when orthologs were present, they were also located in this chromosome. Our search for orthologs revealed a highly variable conservation level in these genes (Fig. 3), which leads us to the conclusion that their β(1-3)-galactosyltransferase function might be specific to only a few species. This variability was not unexpected, as the structures present in the side chains are particular to each species/strain, with different carbohydrates and structural modifications previously described by others [65, 66].

The species that presented the highest number of orthologs from the SCG/R/L family were *L. major* Friedlin (14 genes), *L. infantum* (12 genes), *L. donovani* HU3 (11 genes), *C. fasciculata* (10 genes) and *Leptomonas pyrrhocoris* (7 genes) (Fig. 3). Representatives of *L. pyrrhocoris* do not perform β(1-3)-galactosyltransferase activity [67], while, in several strains of *L. infantum* studied by Coelho-Finamore et al. [66], only glucose was present in the side-chain, with up to three residues found. As such, it is possible that the products of these genes could perform the transfer of other carbohydrates. Another interesting point was that the LV39c5 and SD 75.1 strains of *L. major* did not code the majority of the representatives from the SCG family, even though these strains are closely related to the query used to search for the orthologs (*L. major* Friedlin). However, *L. major* SD 75.1 presents low levels of β1,3 galactosyl residues (scGal) in its LPG side chain [13], which helps explain this result. On the other hand, the long chains of scGal [21, 56] present in *L. major* LV39c5 indicate that this apparent contradiction could be related to the incompleteness of its genome assembly, which comprises 849 scaffolds with total gap length of 404,142 bp (NCBI/Assembly accession no. GCA_000331345.1). The distribution of *SCGR1-6* orthologs was broader in comparison to *SCG1-7* (Fig. 3), despite the β(1-3)-galactosyltransferase activity in LPG of the former not being experimentally proven, as discussed above. All of these genes cluster together with *SCA* family members, implicating their possible role in side-chain modifications that remains thus far unelucidated. The family of *SCG* genes localize in chromosomes 16 and 27 in *C. fasciculata* and *L. pyrrhocoris*, respectively, close to members of the *SCGR/L* and *SCA* families. The fact that this organization is observed in these more basal species provides evidence that homologs of these genes were scattered throughout various chromosomes during the evolution of *Leishmania* species.

### The expression of most, but not all genes that participate in LPG synthesis is constitutive in promastigote and amastigote forms across the four compared *Leishmania* spp

Once the catalog of *Leishmania* LPG-related genes was identified, we investigated whether these were *de facto* expressed, which would support a functional activity. By leveraging the transcriptional profiles available in public databases, we retrieved studies that compared between metacyclic promastigotes and amastigote forms of *L. major* and *L. amazonensis* [37], as well as in cultures of promastigote and amastigote *L. donovani* [35] and *L. mexicana* [36] (see Additional file 2: Table S2 for more information). We focused on these life stages due to relevance to both parasite-sand fly (promastigote) and parasite-mammal host (amastigote) interactions and, although the axenic form is less biologically meaningful compared to the intracellular forms, it is of importance to study expression of these genes under standard, *in vitro* growth. Although *L. amazonensis* was not included in the search for LPG orthologs in our original set of organisms because of poor genome quality (assembled only at the contig-level), we decided to include the RNA-seq data available for this species by mapping its reads against the reference *L. mexicana* genome.

**Fig. 6:**
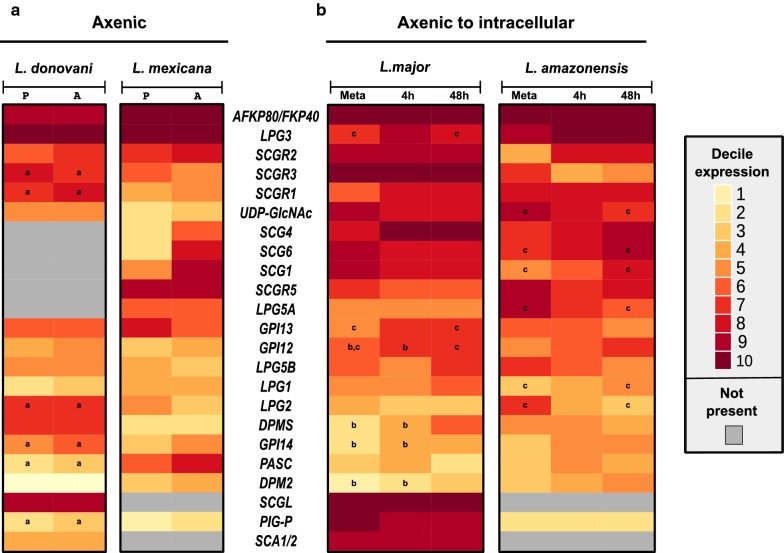
Reanalysis of expression data for genes involved in LPG synthesis in four *Leishmania* species. The expression of LPG genes across four *Leishmania* species (*L. mexicana*, *L. amazonensis*, *L. donovani* and *L. major*) are shown, with each cell representing the expression of a gene (discretized into deciles of cpm values) in a given parasite stage. **a** Axenic cultures of *L. donovani* and *L. mexicana* in promastigote (P) and amastigote (A) forms. **b** Forms of *L. major* and *L. amazonensis* at different life forms/timepoints during the transition of metacyclic promastigotes to intracellular amastigotes at 4 h and 48 h post-infection of macrophages. Genes absent in at least two species are not shown. Differences in expression across stages within each species were evaluated using non-parametric tests (see “Methods”), with lowercase letters indicating significant differences (*P* < 0.05) in the expression of the genes within samples

The expression results were transformed into deciles considering the complete expression set for a given experiment, which unveiled the transcriptional profile of the LPG orthologs (Fig. 6). Similar expression patterns were observed when comparing between life stages or parasite species, indicating that the transcriptional activity of these genes is mostly constitutive. This contrasts with earlier evidence showing that LPG was predominantly expressed in promastigote forms [12]. An explanation for this is that these genes, especially those coding for transferase activities (e.g. *LPG2*, *LPG3*, *LPG5*), also participate in pathways that lead to the synthesis, by amastigote forms, of other membrane compounds, such as PPGs, GIPLs and PGs [68]. On the other hand, initial microarray methods have characterized gene expression in *Leishmania* as mostly constitutive [69], while recent evidence from RNA-seq studies has pinpointed more marked differences between these life stages [36, 70, 71].

Of note, a high-expression cluster formed by *AFKP80/FKP40* and *LPG3* genes (between the 8th and 10th decile) was found in all four species (Fig. 6), indicating a relevant role for the encoded proteins regardless of parasite life stage. High expression levels of the *SCG/R/L* family genes coding for galactosyltransferases were found in *L. major* and *L. amazonensis*, presenting evidence for their coordinated expression. The genes from this family in the same genomic neighborhood (i.e. *SCGRs*, *SCGL*, and *SCAs*) also presented similar expression patterns, which could be related to the polycistronic transcription characteristic of trypanosomatids [72]. The LPG-related genes identified in our genomic data mining approach were found to be expressed across various *Leishmania* species and life stages, in both axenic culture and intracellularly, supporting our genome mining efforts that indicate that the identified orthologs play corresponding biological roles in these parasites.

## Conclusions

Here we present an extensive genomic data mining effort with regard to the repertoire of lipophosphoglycan-related genes in a select group of trypanosomatids. The multiple gene gain/loss events identified during our analysis help to explain some of the observed intra- and interspecies variation of LPG structure. The diversity of LPG has been the focus of many experimental studies in recent years, especially given its importance in host-pathogen and vector-parasite interactions, as well as the vaccine candidate potential of some of the proteins that participate in LPG synthesis. As no previous effort to systematically summarize and evaluate the distribution and relatedness of LPG genes in these organisms has been attempted, our findings contribute towards this objective by allowing a more comprehensive view of the genomic aspects related to the synthesis of LPG.

## Supplementary information

**Additional file 1: Table S1.** Metadata of the genomes included in the study.

**Additional file 2: Table S2.** Metadata of the RNA-seq gene expression studies re-analyzed in the work.

**Additional file 3: Table S3.** Metadata about the genes chosen as query in orthologous searches.

**Additional file 4: Table S4.** Information on re-annotated genes and newly predicted open reading frames.

**Additional file 5: Table S5.** Orthologs genes information (ID, protein products, organism, corresponding open reading frames coordinates and strand).

## Data Availability

Data supporting the conclusions of this article are included within the article and its additional files. The data used in this study are all available from public databases (TriTrypDB (http://www.tritrypdb.org), NCBI/SRA (http://www.ncbi.nlm.nih.gov/sra), NCBI/Gene Expression Omnibus (http://www.ncbi.nlm.nih.gov/geo)) and the relevant sequence accessions used to perform the analyses described in this manuscript are listed in the main text and the accompanying supplementary materials.

## Abbreviations

CL: cutaneous leishmaniasis
d-Ara*p*: d-Arabinopyranose
GIPLs: glycosylinositolphospholipids
KEGG: Kyoto Encyclopedia of Genes and Genomes
GPI: glycosylphosphatidylinositol
LAG: lipoarabinogalactan
LPG: lipophosphoglycan
ML: mucocutaneous leishmaniasis
NSTs: nucleotide-sugar transporters
PG: phosphoglycan repeat
PPG: proteophosphoglycan
scGal: galactosyl residues
VL: visceral leishmaniasis
